# Does a low FODMAPs diet reduce symptoms of functional abdominal pain disorders? A systematic review in adult and paediatric population, on behalf of Italian Society of Pediatrics

**DOI:** 10.1186/s13052-018-0495-8

**Published:** 2018-05-15

**Authors:** Rossella Turco, Silvia Salvatore, Erasmo Miele, Claudio Romano, Gian Luigi Marseglia, Annamaria Staiano

**Affiliations:** 1 Department of Translational Medical Science, Section of Pediatrics, University of Naples “Federico II”, Via S. Pansini 5, 80131 Naples, Italy; 2 Department of Medicine and Surgery, Section of Pediatrics, University of Insubria, Varese, Italy; 3 Endoscopy and Gastroenterology Unit, Department of Pediatrics, University of Messina, Messina, Italy; 4 Department of Science Clinical-Surgery Diagnostic and Paediatric of University of Pavia, Pavia, Italy

**Keywords:** Diet, FODMAPs, IBS, FGIDs, Paediatric, Abdominal pain

## Abstract

**Background:**

Despite the rising of the Functional Gastrointestinal Disorders (FGIDs)’ incidence in the last years, the etio-pathogenesis of FGIDs remains unclear. The diet seems to play an important role in these disorders. Indeed, at least two thirds of adult patients with Irritable Bowel Syndrome (IBS) and of children with FGIDs perceive their GI symptoms to be food-related. In particular, in the last years, more interest has been focused in the low Fermentable Oligosaccharides, Disaccharides, Monosaccharides, and Polyol (FODMAPs) diet.

**Aims:**

To provide a systematic review on the efficacy of a low FODMAPs diet in reducing symptoms associated with functional abdominal pain disorders.

**Methods:**

Cochrane Library, MEDLINE (via Pubmed), and EMBASE databases from inception to June 2017 were searched. We included randomized controlled trials (RCTs), prospective and retrospective studies, systematic reviews and meta-analyses, reporting the efficacy of the FODMAPs diet intervention in FGIDs patients.

**Results:**

Nineteen studies were eligible. A FODMAPs-restricted diet is beneficial in 12/13 intervention trials. The low FODMAPs diet improves overall GI symptoms, especially abdominal pain and bloating. In children, only one study reported positive results of a low FODMAPs diet. No effect was found for the lactose free diet whilst fructose-restricted diet was effective in 3/4 studies. The duration of the intervention was very different among the studies, ranging from 2 days to 16 months, and from 3 and 9 weeks for the RCTs. The majority of the trials presented differences in symptoms scoring scales, diet, food diaries, and food frequencies questionnaire.

**Conclusions:**

The FODMAPs-restricted diet may be an effective dietary intervention for reducing IBS symptoms in adults. In children, there are promising data, although only one randomized double-blind study exists and further data are needed to better clarify the role of FODMAPs and fructose-restricted diet in IBS. The current evidence does not support the use of a lactose-restricted diet in children with FGIDs.

## Background

More and more adults and children complain of abdominal pain, whose origin is in the 95% of functional nature. Abdominal pain is associated to different functional gastrointestinal disorders (FGIDs), among which the irritable bowel syndrome (IBS) is the most common, being reported in 10–25% of adult population [1] and in 0–45% of paediatric population [2]. Despite the rising incidence of FGIDs in the last years, no biomarker or gold standard test is able to prove the diagnosis. The Rome IV criteria for FGIDs, recently updated [3, 4], are currently used in clinical practice to help paediatricians and physicians to identify these disorders. To date, the etio-pathogenesis of FGIDs, and in particular of the IBS, remains unclear although different mechanisms have been proposed. These include increased pain sensitivity or visceral hypersensitivity [5, 6], abnormal gut motility [7], small intestinal bacterial overgrowth [8], low-grade intestinal inflammation [9], psychosocial factors [10] and dysregulated gut–brain axis [11, 12]. Diet and nutrition seem to matter: indeed nutrients can interfere with GI motility, sensitivity, barrier function, and gut microbiota [13] bringing to an atypical modulatory mechanism in the gut. Moreover, it has been reported that at least two thirds of adult patients with IBS [14–16], as well as two thirds of children with functional GI disorders [17], perceive their GI symptoms to be food-related, making dietary management an important tool in the treatment of IBS. In the past years restriction diets were based on the exclusion of a single carbohydrate, such as the lactose or the fructose, in the suspicion of food intolerance. Nevertheless, in the last years, more interest has been focused on the low FODMAPs diet, which comprehensively lowers the intake of several fermentable carbohydrates. The acronym stands for fermentable oligosaccharides, disaccharides, monosaccharides, and polyol (FODMAPs) diet and includes foods with fructose in excess of glucose (pears and apples), oligosaccharides including fructans (wheat and onion), galacto-oligosaccharides (legumes) and sugar polyols such as sorbitol and mannitol (stone fruits and artificial sweeteners), and lactose [10]. The ways through which FODMAPs can lead to GI symptoms are different, including abnormal luminal distension, changes in the gut microbiota, in GI endocrine cells, in immune function, and/or in the intestinal barrier [18–21]. Recently Chumpitazi et al. demonstrated that in paediatric IBS, a low FODMAPs diet decreases abdominal pain frequency [22]. Furthermore, other studies showed promising effect of the low FODMAP diet in reducing functional GI symptoms [23, 24]. However, the evidence of its effectiveness, especially in paediatric age, is limited. The aim of our study was to provide a systematic review of the literature on the efficacy of a low FODMAPs diet in FGIDs.

## Methods

### Search strategy

This review was performed according to a predesigned protocol recommended for systematic review [24]. We conducted a computerized literature search of the Cochrane Library, MEDLINE (via Pubmed), and EMBASE databases from inception to June 2017, with the following search terms: “FODMAP”, “FODMAPs”/ “fermentable oligosaccharides, disaccharides and monosaccharides and polyols”, “fermentable, poorly absorbed, short chain carbohydrates”, “lactose free-diet” and “functional gastrointestinal disorders”, “functional abdominal pain”, “recurrent abdominal pain”, “irritable bowel syndrome”. We did not apply geographical restrictions while we considered only papers written in English language. In addition, the reference lists of all identified articles were examined to identify studies not captured by electronic searches. The electronic search and the eligibility of the studies were assessed independently by 2 of the authors (CR, EM). Differences were discussed, and consensus reached.

### Selection criteria

For inclusion, studies had to involve subjects with IBS and/or FGIDs and had to investigate the efficacy of a FODMAP-restricted diet intervention. No age limits were adopted. Due to the expected paucity of studies, besides all randomized controlled trials (RCTs), we included prospective and retrospective studies, systematic reviews and meta-analyses, reporting the efficacy of the FODMAPs diet intervention in FGIDs patients. Exclusion criteria were language other than English.

### Data extraction and quality assessment

Two investigators (SS and RT) independently extracted, summarized and completed a data extraction form for all the eligible studies. Data from each eligible study were extracted without modification of original data onto custom-made data collection containing items on general information, baseline characteristics of participants, study setting, interventions, and outcomes (Table 1). Disagreements were resolved by consensus with a third reviewer (AS).

**Table 1:** Characteristics of the FODMAPs studies included in the systematic review

Study	Methodology	Participants	Intervention	Duration	Outcome measurements and instruments	Key results	Quality
Bohn 2015 [20]	Randomized, single- blind, trial	Adults aged 18–70 y (*N* = 75) IBS	Low FODMAPs diet	4 wks	Severity of IBS symptoms; Instruments: IBS-SSS	33 (87%) low FODMAP and 34 (92%) traditional IBS diet group completed the study;IBS symptom severity was significantly reduced in both groups compared to baseline; however, the score did not differ between the groups;19 (50%) low FODMAP group and 17 (46%) traditional IBS diet group responded to the interventions	High
Chumpitazi 2015 [22]	Randomized, double-blind, cross-over study, with wash-out	Children aged 7–17 y (*N* = 33) IBS	Low FODMAPs diet	2 days	Children pain episodes frequency; Instruments: Pain and Stoll Diary	17 children began with the TACD, and 16 began with the low FODMAP diet; children had fewer daily abdominal pain episodes during the low FODMAP as compared to the TACD dietary intervention [1.1 ± 0.2 vs. 1.7 ± 0.4 pain episodes per day, respectively; *P* < 0.05].	Moderate
de Roest 2013 [33]	Prospective observational study	Adults (aged non specified) (*N* = 90) IBS	Low FODMAPs diet	6 wks	Improvement of GI symptoms including abdominal pain, bloating, flatulence and diarrhoe; Instruments: GSRS (7-point Likert scale)	90 patients with IBS (47%) completed the whole study. Symptoms significantly improved at follow-up (44% patients with improvement in abdominal pain, 38% in bloating, 38.5% in constipation, 60% in diarrhea). Significant positive correlation between adherence to diet and improvement in individual GI symptoms	Low
Escobar 2014 [45]	Retrospective study	Children and adults aged 2–19 y (*N* = 222) RAP	Low-fructose diet	2 months (not clearly specify)	Improvement of abdominal pain; Instruments: Pain scale score	93 of 121 patients with BTH positive (76.9%) reported resolution of symptoms on a low-fructose diet (*P* < 0.0001) respect to 55 of 101 patients (54.4%) with negative BHT for fructose (*P* = 0.37).	Low
Gijsbers 2012 [42]	Prospective study with DBPC test of provocation	Children aged 4–16 y (*N* = 220) RAP	Low-lactose and/or fructose diet	3 day of provocation test	Disappearance of abdominal pain with elimination, recurrence with provocation and disappearance with re- elimination; Instruments: not specify	Pain disappeared upon elimination in 24/38 patients with lactose malabsorption, and in 32/49 with fructose malabsorption. Open provocation with lactose and fructose was positive in 7/23 and 13/31 patients. DBPC provocation in 6/7 and 8/13 patients, was negative in all. However, several children continued to report abdominal symptoms upon intake of milk or fructose.	Low
Gomara 2008 [43]	Prospective study	Children aged 7–17 y (*N* = 32) FGIDs	Low-fructose and low-sorbitol diet	2 months	Improvement in their GI symptoms; Instruments: not specify	Among the group with positive fructose breath test results, 9 of 11 patients (81%) reported almost immediate improvement in their symptoms; only abdominal pain and bloating were significantly reduced (P < 0.05)	Low
Gremse 2003 [41]	Randomized, double-blind, cross-over study	Children aged 3–17 y (*N* = 30) RAP and lactose maldigestion	Low-lactose diet	2 wks	Improvement in their GI symptoms; Instruments: Symptoms Daily Diary	Significant increase in abdominal pain experienced by study participants during the lactose ingestion period when compared to the lactose-free period	Moderate
Halmos 2014 [28]	Randomized, controlled, single-blind, cross-over trial	Adults aged 23–60 y (*N* = 38) IBS	Low FODMAPs diet	21 days	Improvement in their GI Symptoms; Instruments: Daily symptom scale; VAS	30 IBS participants (91%) and 8 controls (67%) completed the study; 70% IBS subjects had lower overall GI symptom scores on low FODMAP diet compared with typical and subjects’ habitual diet. Similar results with individual symptoms.Bloating, pain, and passage of wind also were reduced while IBS patients were on the low-FODMAP diet, but diarrhea-predominant IBS was the only subtype with altered fecal frequency and King’s Stool Chart scores.	Moderate
Houstoft 2016 [31]	Randomized Double-blind, placebo controlled, cross-over study	Adults aged 18–52 y (*N* = 20) diarrhea-predominant or mixed IBS	Low FODMAPs diet	9 wks	Improvements in GI symptoms; Instruments: IBS-SSS	There was a significant improvement in all IBS symptoms after 3 weeks of LFD with a mean reduction in IBS-SSS total score of 163.8. When supplementing the LFD with FOS or placebo, significantly more participants reported symptom relief in response to placebo (80%) than FOS (30%; *P* = .013).	Moderate-High
Lebenthal 1981 [40]	Prospective study	Children aged 6–14 y (*N* = 69) RAP	Low-lactose diet	12 months	Improvement in RAP; Instruments: Symptoms Diary	After 12 months of elimination diet symptoms of RAP resolved in 6/15 (40%)lactose malabsorbers, 5/13 (38,4%) lactose absorbers and in 5/12 (41.7%) lactose absorbers in regular diet	Low
Maagard 2016 [36]	Retrospective study	Adults aged 18–85 y (*N* = 180) IBS and IBD	Low FODMAP diet	16 months	Improvement of symptoms and stool pattern; Instruments: Questionnaire, IBS SSS, stool pattern	Eighty-six per cent of patients on LFD reported either partial (54%) or full (32%) efficacy with greatest improvement of bloating (82%) and abdominal pain (71%). After dietary intervention, the proportion of patients producing normal stools increased, with 41% in the IBS group (P < 0.001)	Low
Ong 2010 [30]	Randomised, single-blind, cross-over study	Adults aged 22–68 y (N = 30) IBS	low FODMAPs-diet	2 days	Improvement of symptoms; Instruments: GI symptoms questionnaire; food diares; breath test	All symptoms were significantly worsened with high FODMAP diet in patients with IBS. Dietary FODMAPs induce prolonged hydrogen production in the intestine that is greater in IBS patients	Moderate-High
Pedersen 2014 [29]	Randomised unblinded controlled trial	Adults aged 18–74 y (*N* = 123)	Low FODMAPs diet	6 wks	Changes in IBS symptoms and quality of life; Instruments: IBS-SSS and IBS-QoL	Overall there was a significant reduction of IBS-SSS mean ± SD in all patients from baseline to week 6, mean IBS-SSS score 77 ± 104, P < 0.01, as well as in each treatment group (LFD, *P* < 0.001, LGG, *P* < 0.01 and ND, *P* = 0.03).At week 6, comparing mean IBS-SSS between all three groups, a statistically significant reduction in the IBS-SSS was observed in LFD and LGG groups compared to the ND group, mean IBS-SSS 133 ± 122 vs 68 ± 107, 133 ± 122 vs 34 ± 95, P < 0.01	Moderate-High
Pedersen 2014 [32]	Prospective, uncontrolled pilot study	Adults aged 18–74 y (*N* = 19) IBS	Low FODMAPs diet	6 wks	Changes in IBS symptoms and quality of life; Instruments: IBS-SSS and IBS-QoL	All 19 patients with IBS completed the study.Significant improvement in IBS in control period and following dietary intervention period.Low FODMAP diet further reduced symptoms (11 patients [57%] improved to mild IBS severity). Significant IBS-QoL change during low FODMAP diet period	Moderate
Staudacher 2011 [21]	Prospective, controlled, study	Adults aged 26–50 y (*N* = 82) IBS	Low FODMAPs diet	Unclear	Improvement of GI symptoms: Instruments: validated IBS Global Improvement Scale (7-point Likert scale); Four statements on satisfaction with symptom response and dietary advice	Significantly more patients in the low FODMAP group compared to the standard group reported improvements in bloating (low FODMAP 82% versus standard 49%, *P* = 0.002), abdominal pain (low FODMAP 85% versus standard 61%, *P* = 0.023) and flatulence (low FODMAP 87% versus standard 50%, *P* = 0.001). There were no significant differences in the proportion of patients reporting improvement in constipation between groups.	Moderate-Low
Staudacher 2012 [27]	Randomized, controlled tiral	Adult aged 18–65 y (*N* = 41) IBS	Low FODMAPs diet	4 wks	Improvement of GI symptoms; Instruments: symptom diary based on the GI Symptom Rating Scale	All 41 patients were included for ITT and 35 in the PP analysis.At follow-up, more patients inthe intervention group reported adequate symptom control with ITT (68% vs 23%) and for PP (81% vs 26%)	Moderate
Valeur 2016 [35]	Prospective study	Adults aged > 18 y (*N* = 63) IBS	Low FODMAPs diet	4 wks	Decreased GI symptoms and evaluation of Short-chain fatty acids (SCFAs); Instruments:IBS-SSS	Sixty-three patients completed the study. Following the dietary intervention, IBS-SSS scores improved significantly (*p* < 0.0001). Total SCFA levels were reduced in fecal samples analyzed both at baseline (*p* = 0.005) and after in vitro fermentation for 24 h (*p* = 0.013).	Low
Wildersmith 2017 [34]	Prospective study	Adults aged 26-58y (*N* = 653) FGIDs	Low FODMAPs diet	6–8 wks	Decreased global symptoms; Instruments: A nonstandard questionnaire on abdominal symptoms (10-point Likert scales) Bowel and dietary habits	237 of 312 (76%) patients completed the studyOver 80% of patients attained adequate global symptom relief; 93 and 96% of patients with fructose or lactose malabsorption, respectively, 85% adequate relief in patients with diarrhea, 96% with bloating, and 51% with constipation	Moderate
Wintermeyer2012 [44]	Prospective study	Children aged 3–14 y (*N* = 75) RAP	Low-fructose and low-sorbitol diet	4 wks	Improvement frequency and intensity of abdominal pain; Instruments: nonstandard questionnaire	A median decline of weekly pain frequency from 4 (mean 3.64 + 1.6) before diet to 1 (mean 1.46 + 1.4; *p* < 0.001) under fructose restriction was documented. The intensity of pain decreased from median 6 (mean 5.83 + 2.0) before intervention to median 3 (mean 3.4 + 2.5; p < 0.001) with diet. Several additional life quality-influencing parameters such as daily stool frequency, nausea, problems to fall asleep, missed school days also improved significantly.	Moderate

*IBS* irritable bowel syndrome, *RAP* recurrent abdominal pain, *TACD* typical American childhood diet, *LFD* low FODMAP diet, *ITT* intention-to-treat, *PP* per protocol

The same authors (RT, SS), independently, assessed the risk of bias of the included studies via the Cochrane risk of bias tool [25, 26] for the follow domains: random sequence generation (selection bias), allocation concealment (selection bias), blinding of participants and personnel (performance bias), blinding of outcome assessment (detection bias), incomplete outcome data (attrition bias) and selective reporting (reporting bias) (Table 2). As “other bias” we included the lack of a control group (bias in design). For each outcome, review authors’ judgments were categorized as “low risk,” “high risk” or “unclear risk of bias.” Discrepancies were resolved by discussion with a third reviewer (AS). The quality of systematic reviews and meta-analyses were evaluated by the PRISMA recommendation (Table 3).

**Table 2:** For each domain risk of bias is rated as high, unclear or low

Reference	Random sequence generation (Selection bias)	Allocation concealment (Selection bias)	Blinding of participant and personnel (Performance bias)	Blinding of outcome assessment (Detection bias)	Incomplete outcome data (Attrition bias)	Selective reporting (Reporting bias)	Choice of control groups (Bias in design)
Bohn 2015 [20]	Low risk	Unclear risk	Low risk	Low risk	Low risk	Low risk	Low risk
Chumpitazi 2015 [22]	Low risk	Low risk	Low risk	Low risk	Low risk	Low risk	High risk
de Roest 2013 [33]	No randomised	High risk	High risk	High risk	High risk	High risk	High risk
Escobar 2014 [45]	High risk	High risk	High risk	High risk	High risk	High risk	Low risk
Gijsbers 2012 [42]	High risk	High risk	High risk	High risk	High risk	High risk	High risk
Gomara 2008 [43]	High risk	High risk	High risk	High risk	High risk	High risk	High risk
Gremse 2003 [41]	Unclear risk	Unclear risk	High risk	High risk	High risk	High risk	Low risk
Halmos 2014 [28]	Low risk	Unclear risk	Low risk	Unclear	Low risk	Low risk	High risk
Houstoft 2016 [31]	Low risk	Low risk	Low risk	High risk	Unclear	Low risk	High risk
Lebenthal 1981 [40]	High risk	High risk	High risk	High risk	High risk	High risk	High risk
Maagard 2016 [36]	High risk	Unclear risk	High risk	Unclear	High risk	Low risk	High risk
Ong 2010 [30]	Low risk	Unclear risk	Low risk	Unclear	Low risk	Low risk	Low risk
Pedersen 2014 [29]	Low risk	Low risk	High risk	High risk	High risk	High risk	High risk
Pedersen 2014 [32]	High risk	High risk	High risk	High risk	High risk	Low risk	High risk
Staudacher 2011 [21]	Unclear	High risk	High risk	High risk	Unclear	High risk	High risk
Staudacher 2012 [27]	Low risk	Low risk	Low risk	High risk	High risk	High risk	Low risk
Valeur 2016 [35]	High risk	High risk	High risk	High risk	High risk	Low risk	High risk
Wildersmith 2017 [34]	High risk	High risk	High risk	High risk	low risk	Low risk	High risk
Wintermeyer 2012 [44]	High risk	High risk	High risk	High risk	High risk	High risk	High risk

**Table 3:** Evaluation of systemaic reviews and Cochrane review

Study	Study design	Population	Kind of studies	Number of studies	Number of participants	Abstracts	Objective	Protocol	Search	Assessment risk of bias in included studies	Assesment of risk of bias across studies	Discussion	Funding
Marsh 2016 [23]	Systematic review and meta-analysis	Adult and children	RCTS and non RCTs	6 RCTs + 16	723 (only 33 children)	Yes	Yes	Yes	Yes	Yes	Unclear	Yes	Not reported
Newlove-Delgado 2017 [24]	Cochrane review	Children (5–18 years)	RCTs	19 (only 1 with FODMAPs e 1 with fructose-restricted diet)	1453 (137 with FODMAPs or fructose diet)	Yes	Yes	Yes	Yes	Yes	Yes	Yes	Reported
Rutten 2015 [37]	Systematic review	Children (3–18 years)	RCTs	24 (only 2 with lactose free diet)	1390 (116 on lactose free diet)	Yes	Yes	Yes	Yes	Yes	Unclear	Yes	Reported

## Results

Nineteen full-text studies [20–22, 27–36, 40–45] and 3 systematic reviews and meta-analyses [23, 24, 37] were included in the final analysis according to the selected criteria. Among the studies, 7 concern paediatric age.

### Paediatric studies

Two Cochrane Reviews [23, 24] evaluating the effect of FODMAPs diet in paediatric age analysed the randomized double-blind, crossover trial by Chumpitazi et al. [22]. This study included 33 IBS children coming from Texas (all fulfilling the IBS Rome III criteria: 24 with IBS-Constipation, 3 with IBS-unsubtyped, 3 with IBS-Mixed and 3 with IBS-Diarrhoea), 67% of which were female with a mean age of 11.5 ±3.0 years. After one-week baseline period, children were randomized to receive either a low FODMAPs diet containing 0.15 g/kg/day (maximum 9 g/day) of FODMAPs or a typical American childhood diet (TACD) contained 0.7 g/kg/day (maximum 50 g/day) of FODMAPs. The intervention lasted for 48 h, followed by a 5-day washout period before crossing over to the other diet for other 48 h. The frequency and the characteristics of abdominal pain and of the other GI symptoms were evaluated through a Pain and Stool Diary. Stool samples were also collected to assess the microbiome composition and metabolic capacity. The results showed fewer episodes of abdominal pain among children on low FODMAPs diet respect to children on TACD [1.1 ± 0.2 vs. 1.7 ± 0.4 pain episodes per day, respectively; *P* < 0.05]. When authors compared the data following diet respect to baseline, they observed fewer daily abdominal pain episodes during the low FODAMPs diet (*p* < 0.01) but more episodes during the TACD (*p* > 0.01). Authors found that responders (children who had significant improvement on the low FODMAPs diet only) at baseline were enriched in taxa with known greater saccharolytic metabolic capacity (e.g. Bacteroides, Ruminococcaceae, Faecalibacterium prausnitzii) and three Kyoto Encyclopedia of Genes and Genomes orthologues, of which two relate to carbohydrate metabolism. Deep analysis of microbiota composition and structure revealed no change in α-diversity (number of operational taxonomic units (OTU), ie, number of species) in children after a one-week low FODMAPs diet.

### Adult studies

Marsh et al. [23] included 21 adult studies assessing the effect of the FODMAPs diet on functional gastrointestinal symptoms. In a RCT, Staudacher et al. [27] compared the effects of a 4 weeks fermentable carbohydrate restriction diet to a habitual diet on luminal microbiota, SCFA, and GI symptoms. The study subjects included 41 patients with IBS, defined using Rome III criteria, aged 18–65 years old. At baseline, no significant differences were found between the low FODMAPs diet (7/19, 37%) and the habitual diet group (9/22, 58%; *P* = 0.79) in response to the global symptom question. However, at follow-up, more patients in the low FODMAPs diet group reported adequate symptom control compared with the habitual diet group, when analysing the intention-to-treat (13/19, 68% vs. 5/22, 23%; *P* = 0.005) and per protocol (13/16, 81% vs. 5/19, 26%; *P* = 0.002) data. About the incidence and severity of symptoms, at baseline, there wasn’t a significantly difference between groups, except for nausea, which was less frequent and less severe in the low FODMAPs diet group. However, at follow-up, there was a lower incidence of bloating, abdominal pain, and overall symptoms and a lower mean daily severity scores in the low FODMAPs diet group. Diarrhoea severity scores were similar in both groups at follow up. Stool frequency and consistency were similar at baseline, but at follow-up, after adjusting data for baseline, lower stool frequency and a greater proportion of stools with normal consistency were observed in low FODMAPs diet group rather than in the habitual diet group. The total luminal bacteria at follow-up did not differ between groups; however, there were lower concentrations (*P* < 0.001) and proportions (P < 0.001) of Bifidobacteria in the intervention group compared with controls, when adjusted for baseline.

In 2014 Halmos et al. [28] performed a randomised, controlled, single blind cross-over study on 38 Australian subjects. Subjects included 30 IBS patients defined according to Rome III criteria, of whom 10 with IBS-D, 13 with IBS-C, 5 with IBS-M and 2 with IBS-U (70% female, mean age of 41 years) and 8 healthy adults. Participants were randomised to receive either a high or low FODMAPs diet for 21 days. Before trying the next diet, subjects’ symptoms were needed to return to the same level as during the baseline period. Stool samples were also collected for 5 days at the end of each diet period to evaluate the faecal consistency, frequency and weight. IBS subjects showed lower gastrointestinal symptom scores while on low FODMAPs diet (22.8 mm on the VAS) rather than on Australian diet (44.9 mm on the VAS; *p* < 0.01). The improvement in GI symptoms scores was observed in 70% of IBS patients. On the contrary the healthy subjects did not present significantly differences in GI symptoms scores during the two diets. Regarding stool characteristics, the only significant differences were a lower King’s Stool Chart score and reduced stool frequency on the low FODMAPs diet compared with the typical Australian diet in IBS-D subtype.

In the same year, Pedersen et al. [29] performed a randomised, un-blinded controlled trial on the effect of a low FODMAPs diet (LFD), compared with the use of a Lactobacillus rhamnosus GG (LGG) capsule and with a normal Danish/Western diet (ND). Subjects consisted of 123 IBS patients defined by Rome III criteria, 73% of whom were female, with a mean age of 37 years. Subjects were randomly divided in 3 diet groups: the LFD group including 42 subjects, the LGG group including 41 subjects and the ND group including 40 subjects. The intervention lasted 6 weeks*.* Subjects on LFD diet were instructed on the diet by a dietician and a nutritionist, making their FODMAPs intake not quantifiable. Both IBS severity score system (IBS-SSS) and IBS quality of life (IBS-QOL) were evaluated. The authors observed an overall reduction in mean ± SD of IBS-SSS from baseline to week 6 for each group: (LFD, *P* < 0.001, LGG, *P* < 0.01 and ND, *P* = 0.03). Adjusted linear regression analysis of changes of IBS-SSS from baseline covariates toward the study period of 6-weeks in all three groups, showed a statistically significant improvement of IBS-SSS in LFD group vs ND group, (IBS-SSS = 75; 95%CI: 126–24, P < 0.01), but not in LGG group vs ND group, (IBS-SSS = 32; 95%CI: 80–18, *P* = 0.20). Regarding to the IBS-QOL, the authors didn’t find significantly differences among groups (mean ± SD in LFD 8 ± 18 vs. LGG 7 ± 17, LFD 8 ± 18 vs. ND 0.1 ± 15, *P* = 0.13). Analysing the results by subtypes, a significant reduction in the mean IBS-SSS from baseline to week 6 was observed regarding the IBS-D subtype in the 3 diet groups (*p* < 0.01), as well as for the IBS-A subtype in the LFD (*p* = 0.01) and LGG group (*p* = 0.04) but not in the ND group (*p* = 0.12). Instead no significant reduction of IBS-SSS was found in patients with IBS-C type in any diet group.

More recently, Bohn et al. [20] analysed data deriving from a randomized, multicentre single-blind trial, on the comparison between a low FODMAPs diet and a traditional diet in Swedish subjects. Seventy-five IBS patients according to Rome III criteria were randomly assigned to one of the two diets for 4 weeks. The patients on low FODMAPs diet were instructed to which foods to avoid or to ingest while patients on traditional diet were instructed especially on how and when to eat rather than on what to eat.

The IBS Symptom Severity Scale and a 4-day food diary before and at the end of the intervention were recorded. Data showed that the severity of IBS symptom was significantly reduced in both groups compared to baseline (*p* < .0001) without a significant difference between the groups (*p* = 0.62).; however, 19 IBS patients (50%) on low FODMAPs diet and 17 (46%) IBS patients on traditional diet responded to the interventions, without any significantly difference between the groups. Regarding the food diary, at baseline both groups had similar intake of nutrients, and a clear change in dietary intake during the 4 weeks study period was observed in both groups.

Another randomized single blind cross-over study [30] evaluated the effect of a FODMAP-restricted diet in relation to the production of hydrogen and methane and to the possible induction of functional GI symptoms. The authors investigated 15 healthy subjects and 15 subjects with IBS according to Rome III criteria (87% female, median age 41 years) in Australia. Among the 15 IBS subjects, 4 had IBS-D, 7 had IBS C, 2 had IBS-M and 2 had un-typed IBS (IBS-U). After a 7-days baseline period, subjects received either a FODMAP-restricted diet (9 g/day) or a high FODMAPs diet (50 g/day) for 2 days each with a 7 days wash-out period between diets. Gastrointestinal symptoms were evaluated and breath samples were collected on day 2 of each diet. In IBS subjects, all symptoms were significantly worse while on high FODMAPs diet (abdominal pain (*P* = 0.006), bloating (*P* = 0.002), passage of gas (P = 0.002), nausea (*P* = 0.01), heart burn (*P* = 0.025) and lethargy (*P* = 0.012)), while in healthy subjects was recorded only a significant reduction of the passage of gas (*P* = 0.007). No differences were observed for the other symptoms in healthy individuals while on the different diets. Higher levels of breath hydrogen were produced with the high FODMAPs diet respect to the low FODMAPs diet for both groups (*P* < 0.0001). IBS subjects produced higher levels of hydrogen during each dietary period than the healthy subjects (*P* < 0.05).

One prospective controlled trial [21] and one double-blind placebo controlled cross over trial [31] also evaluated the improvement of the IBS symptoms on low FODMAPs diet. Both studies utilized validated questionnaires to study the outcome, the IBS Global improvement scale [21] and the IBS-SSS questionnaire [31] and advices about a FODMAPs diet. IBS patients of both study recorded an improvement of their symptoms.

The other studies were “prospective but uncontrolled” [32–35] or retrospective [37], Table 1.

### Low lactose, sorbitol fructose diet

Rutten et al. [37] included a Cochrane Review [38] evaluating 2 trials about a lactose-free diet in children with recurrent abdominal pain [39, 40]. Lebenthal et al. [40] analysed 38 out of 69 enrolled children with abnormal lactase activity receiving 6 weeks of lactose containing or lactose-free infant formula. Children were divided in two groups, one of lactose malabsorbers (*n* = 21) and the other of lactose absorbers (*n* = 17), according to the lactose tolerance. Increased symptoms were described in 48% of the lactose malabsorbers and 24% of the lactose absorbers after lactose intake; however, *P* values were not reported. Forty of the 69 children continued with a 12-month lactose free diet. Improvement of abdominal pain after 12 months was similar in both groups (40% vs 38%).

In 2003, Gremse et al. [41] performed a randomized, double-blind, cross-over study assessing whether the ingestion of lactose was associated with GI symptoms in 30 children, between 3 and 17 years old, affected by recurrent abdominal pain and lactose maldigestion. Authors found that the symptom scores for abdominal pain, bloating, flatulence, and diarrhoea were similar for subjects with either > 10-ppm or > 20- ppm increase in breath hydrogen testing after lactose. As a matter of fact the mean abdominal pain score during ingestion of lactose-containing versus lactose-free milk was 8.5 ± 3.0 versus 8.5 + 2.2 for subjects with an increase in breath hydrogen concentration of > 20 ppm and 8.2 + 2.3 versus 5.9 ± 1.7 for those with a 10- to 20-ppm increase. One prospective study on the effect of low lactose and/or fructose diet [42], two on the effect of a low fructose and sorbitol diet [43, 44] and one retrospective study [45] on the effect of a low-fructose diet exist in paediatric age (Table 1).

## Discussion

This systematic review includes 19 studies ranging from very low to high methodological quality. Some of the evidences suggest beneficial effects of FODMAPs restricted diet in both adult and children with IBS [22, 27–30]. Differently, no effects of the lactose-restricted diet have been extrapolated in children, while available evidence is promising in supporting the benefit of the fructose-restricted diet in paediatric age.

Dietary intervention is considered as an important non-pharmacological treatment of FGID, especially in the IBS. Recent guidelines consider the diet and the lifestyle advice as the first-line approach in the dietary management of IBS in adults [46]. However, patients with IBS often self-initiate dietary interventions without any specific advice, leading to an increased risk of nutritional inadequacy. This happen because many patients consider their symptoms to be meals-related. In the past, the most common diets for IBS patients focused on restriction of fibres, caffeine, alcohol, and fat [47], but in the last 10 years the low FODMAPs diet was considered as a newcomer to dietary management of IBS and an amount quantity of evidence about the mechanisms and the clinical efficacy of this new diet spread. Dietary carbohydrates can be classified into sugars, oligo-saccharides and polysaccharides, based on their degree of polymerisation. FODMAPs are a discrete group of carbohydrates described as ‘fermentable’ owing to their availability for fermentation in the colon, which is either due to the absence, or reduced concentration, of suitable hydrolase enzymes for digestion (for example, lactase deficiency), or in the case of monosaccharides because of incomplete absorption in the small intestine. These poorly absorbed short-chain carbohydrates include: fructose and lactose, fructans, galacto-oligosaccharides, and polyols or sugar alcohols. Despite their health effects such as increasing stool bulk, enhancing calcium absorption, modulating immune function, and selective stimulation of some microbial, FODMAPs can trigger specific gastrointestinal symptoms in patients with IBS. The presence and the degree of abdominal symptoms vary on the degree of malabsorption experienced by each individual. Short-chain fermentable carbohydrates might exacerbate IBS symptoms through various mechanisms, such as increased small intestinal water volume, colonic gas production and intestinal motility. FODMAPs are indeed poorly absorbed in the small intestine leading to gas production and increase of intestinal osmolarity due to their rapid fermentation and osmotic action. A study about ileostomates has shown that FODMAPs diet increased the fermentable load and volume of liquid delivered to the proximal colon [48]. Short-chain fermentable carbohydrates are also rapidly fermented by the colonic microbiota, resulting in luminal distension and pain in those with visceral hypersensitivity. Moreover, fermentable carbohydrates seem to have an effect on motility as shown by a scintigraphy study demonstrating that fructose–sorbitol ingestion reduced oro-cecal transit time by just over 3 h in healthy people [49].

The present systematic review shows that adherence to a low FODMAPs diet results in improvement of overall functional GI symptoms. As a matter of fact, a significant beneficial effect of a low FODMAPs diet on clinical symptoms was reported by all the studies analysed except for the recent randomized single-blinded trial by Bohn et al. [20] whose studied subjects responded to the intervention regardless of the type of diet (low FOODMAP vs traditional diet). In particular, abdominal pain and bloating, considered as the most troublesome and frequent symptoms in IBS, were highly relieved after FODMAPs diet. Therefore, it is likely that a low FODMAPs diet may be beneficial for the majority of patients suffering by IBS. Moreover it has been demonstrated that most patients with IBS found the diet easy to adhere [33, 34] with improvement of symptoms and quality of life seen in those with the best adherence [50]. A low FODMAPs diet resulted also in an improvement of stool frequency and consistency, except in the study by Staudacher et al. [21], where no differences were found comparing a low FODMAPs diet with a standard diet. In the paper by Pederson et al., the authors showed that the effect of the low FODMAPs diet is dependent upon the IBS subtype, being most effective in patients with the IBS diarrhoeal type [29]. Data regarding the low FODMAPs diet in paediatric age are still missing even if this kind of diet is considered an emerging approach in the dietary management of IBS children [51]. The paper by Chumpitazi et al. [22] appears to be the first looking at the efficacy of this diet in IBS children, showing a decreasing of abdominal pain frequency in children following a low FODMAPs diet respect to children following a typical American childhood diet. The authors also showed that both baseline gut microbiome composition and microbial metabolic capacity could influence the FODMAPs diet efficacy determining the rate of responders and non-responders. Indeed, at baseline responders showed bacteria with greater saccharolytic capacity (such as genera Bacteroides, Clostridiales and family Erysipilotrichaceae) than those who did not respond to the diet. Different studies have investigated the effect of the low FODMAPs diet on the gut microbiota [22, 27, 52, 53]. Staudacher et al. [27] demonstrated that a low FODMAP diet significantly reduces luminal bifidobacteria after 4 weeks in adult patients and suggest the use of prebiotic or probiotic when the low FODMAP diet is followed in the long term. Recently, Holmos [54] et al. showed lower absolute Bifidobacteria concentration, F. prausnitzii and Clostridium cluster IV accompanied by a substantially lower total bacterial load of 47% during the low FODMAPs diet compared with habitual diet. However, the role of microbiota in the low FODMAPs diet is far to be clarified due to methodological problems, influence of confounding factors and large differences between studies.

Historically, in paediatric age, carbohydrate malabsorption has been focused mainly on lactose and fructose. Malabsorption and intolerance to carbohydrates such as fructose and lactose are believed to cause symptoms such as bloating, diarrhoea, and abdominal pain [15] but it is no clear whether lactose or fructose malabsorption are the major basis for chronic gut symptoms in a proportion of patients with FGID. Gijsbers et al. [42] did not demonstrate an intolerance to lactose or fructose in a cohort of 220 children with recurrent abdominal pain (RAP) and observed that despite negative double-blinded placebo-controlled provocation, some children still complained abdominal symptoms when using milk or fructose-containing food. Lactose free diet did not ameliorate symptoms in a cohort of children with RAP [40], while in a randomized double-blind cross over study on 30 children with RAP a significant increase of severity of abdominal pain was recorded during lactose ingestion period respect to the lactose-free period [41]. Data in adult studies are also conflicting, keeping unresolved the dilemma whether the lactose malabsorption is part of the IBS symptoms or the two conditions may simply coexist in some patients. Regarding the fructose free diet, observational studies analysed in the present systematic review reported symptom improvement when children with fructose malabsorption and RAP [44, 45] or FGID [43] were on a fructose-free diet. These results even if promising derived from un-controlled non-randomized studies. Interestingly, very recently, a randomized placebo-controlled cross-over trial [55] of 23 children with IBS showed that pain frequency and bloating were significantly higher during the fructan intervention as compared with the placebo (maltodextrin) intervention. However, more studies are needed to clarify the role of the lactose and fructose restricted diet in the management of IBS children.

The present systematic review is not without limitations. The lack of standardization among studies certainly represents the main problem. Indeed, differences in diet, food diaries, food frequencies questionnaire, duration of the intervention and scoring scale were encountered. The duration of the intervention was very different among studies, ranging from 2 days to 16 months, and from 3 and 9 weeks for the RCTs. Considering that IBS is usually a chronic, sometimes life-long condition with periods of remission and exacerbation, a short duration of the intervention may not be able to catch long-term effect and the real efficacy of the diet intervention. Recently, indeed, a minimum length of 6 months has been recommended to establish long-term efficacy of an intervention [56]. In addition, even if the common primary outcome measure was changes in GI symptoms, the majority of the studies used different scoring scales with few studies using validated questionnaires [20, 29, 31, 32, 34, 36]. Standardization in the use of a unique validated questionnaire to investigate the symptoms’ relief is highly desirable in future studies. Finally, the quality of evidence has to be considered low. Indeed, among all the analysed studies, only 4 were RCTs, and only one was double-blind. Other two double blind randomized studied were not controlled. As a matter of fact, all the studies presented a high risk of bias.

## Conclusion

This systematic review shows that restriction of FODMAPs may be an effective dietary intervention for reducing IBS symptoms in adults. In children, even if data are very promising, just one randomized double-blind study exists and further studies are needed to better clarify the role of FODMAPs. The current evidence does not support the use of a lactose restricted diet in children with IBS, while further studies are needed to establish the role of the fructose restricted diet in the IBS symptoms’ relief in children.

## Abbreviations

FGIDs: Functional gastrointestinal disorders
FODMAPs: Fermentable oligosaccharides, disaccharides, monosaccharides, and polyol
IBS: Irritable bowel syndrome
